# Quantitation of zolpidem in biological fluids by electro-driven microextraction combined with HPLC-UV analysis

**DOI:** 10.17179/excli2018-1140

**Published:** 2018-04-23

**Authors:** Saeid Yaripour, Ali Mohammadi, Isa Esfanjani, Roderick B. Walker, Saeed Nojavan

**Affiliations:** 1 Department of Drug and Food Control, Faculty of Pharmacy, Tehran University of Medical Sciences, Tehran, Iran; 2 Department of Pharmaceutical and Food Control, Faculty of Pharmacy, Urmia University of Medical Sciences, Urmia, Iran; 3 Nanotechnology Research Centre, Faculty of Pharmacy, Tehran University of Medical Sciences, Tehran, Iran; 4 Faculty of Pharmacy, Rhodes University, Grahamstown 6140, South Africa; 5 Faculty of Chemistry, Shahid Beheshti University, Tehran, Iran

**Keywords:** biological fluids, electromembrane extraction, HPLC-UV, quantitation, zolpidem

## Abstract

In this study, for the first time, an electro-driven microextraction method named electromembrane extraction combined with a simple high performance liquid chromatography and ultraviolet detection was developed and validated for the quantitation of zolpidem in biological samples. Parameters influencing electromembrane extraction were evaluated and optimized. The membrane consisted of 2-ethylhexanol immobilized in the pores of a hollow fiber. As a driving force, a 150 V electric field was applied to facilitate the analyte migration from the sample matrix to an acceptor solution through a supported liquid membrane. The pHs of donor and acceptor solutions were optimized to 6.0 and 2.0, respectively. The enrichment factor was obtained >75 within 15 minutes. The effect of carbon nanotubes (as solid nano-sorbents) on the membrane performance and EME efficiency was evaluated. The method was linear over the range of 10-1000 ng/mL for zolpidem (R^2 ^>0.9991) with repeatability ( %RSD) between 0.3 % and 7.3 % (*n* = 3). The limits of detection and quantitation were 3 and 10 ng/mL, respectively. The sensitivity of HPLC-UV for the determination of zolpidem was enhanced by electromembrane extraction. Finally, the method was employed for the quantitation of zolpidem in biological samples with relative recoveries in the range of 60-79 %.

## Introduction

**Figure 1:**
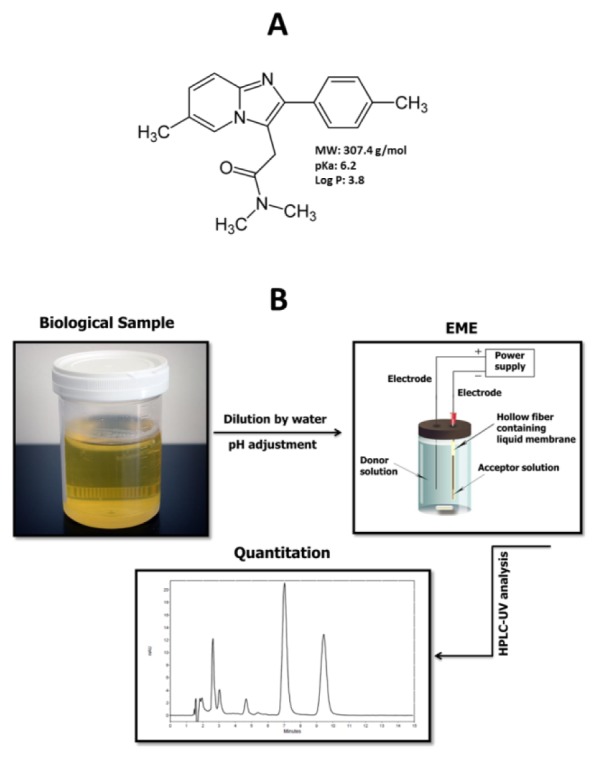
(A) Chemical structure of zolpidem, and (B) schematic illustration of EME-HPLC-UV analysis

Zolpidem (N,N,6-trimethyl-2-(4-methylphenyl)imidazo[1,2-a]pyridine-3-acetamide) (Figure 1A(Fig. 1)) (O'Neil et al., 2001[30]) is a short-acting sedative-hypnotic molecule with a rapid onset of action that is widely prescribed for the treatment of insomnia and other short-term sleeping disorders. Zolpidem is a selective agonist of the omega-1 receptor at the CNS (central nervous system) gamma amino butyric acid (GABA)/chloride channel complex. It is a non-benzodiazepine imidazopyridine which also exhibits a weak anticonvulsant effect (Langtry and Benfield, 1990[21]; Wagner and Wagner, 2000[42]). It was reported that zolpidem is one of the most commonly abused drugs (Hsu et al., 2014[16]), which exhibits important adverse events such as anterograde amnesia and hallucinations. These conditions are enhanced by the alcohol abuse and are important in forensic toxicology. Therefore, the Society of Forensic Toxicologists (SOFT) has listed them as conditions for drug-facilitated sexual assault (DFSA) substances (Hsu et al., 2014[16]; Society of Forensic Toxicologists, 2016[36]; Bosman et al., 2011[5]; Villain et al., 2004[39]). Peak plasma concentrations of zolpidem are observed within 0.5 and 3 h following the oral administration. The major metabolites of zolpidem are pharmacologically inactive and so that they are excreted in urine (56 %) and faeces (37 %), but < 1 % is excreted as unchanged drug. The therapeutic concentration range in the serum is 80-150 ng/mL (Moffat et al., 2011[25]). Therefore, the quantitation of zolpidem in biological samples is important for pharmacokinetic, forensic, and toxicological studies. 

Biological and pharmaceutical samples are complex matrices that often contain salts, metals, proteins, and other macromolecules and organic compounds which may interfere with the analyte of interest when doing instrumental chemical analysis. Therefore, extraction is necessary to isolate and enrich the desirable analyte in complex matrices. In recent years, micro-extraction methods have been developed for the extraction of pharmaceuticals from biological fluids. Electromembrane extraction (EME) of drugs from biological fluids was recently introduced as a new sample preparation technique (Pedersen-Bjergaard and Rasmussen, 2006[32]). In this approach, the target analytes are transported from an aqueous sample, into a supported liquid membrane (SLM) and then into an aqueous acceptor solution placed inside the lumen of a hollow fiber. The charged analyte species is obtained by adjusting the pH of the donor and acceptor fluids. The supported liquid membrane is prepared by an impregnated hollow fiber with the aid of an organic solvent. The driving force for the molecule movement is an electric potential difference (voltage) which provides high analyte extraction in a short time without the need to use other time consuming and expensive procedures. Therefore, EME is a good sample preparation technique for the extraction of drugs from biological fluids in pharmacokinetic and toxicological studies. This process is compatible with conventional analytical equipment (HPLC, GC, CE, etc.). The limits of detection and quantitation of an analytical method can be improved by enrichment of the analyte concentration which is obtained by EME technique. Furthermore, using low quantities of an organic solvent (µL), EME is environmentally friendly (Gjelstad and Pedersen-Bjergaard, 2011[11]; Marothu et al., 2013[24]; Huang et al., 2015[17]). To date, EME has been used for the extraction of drugs (Huang et al., 2015[17]; Davarani et al., 2013[6]; Oliveira et al., 2017[31]; Nojavan et al., 2012[28]; Asadi and Nojavan, 2016[2]), dyes (Nojavan et al., 2013[29]; Yaripour et al., 2016[45]), heavy metals (Kubáň et al., 2011[20]), environmental pollutants (Villar-Navarro et al., 2016[41]), amino acids, and peptides (Strieglerová et al., 2011[38]; Balchen et al., 2011[3]; Nojavan et al., 2016[27]) from different sample matrices. 

Several analytical methods such as capillary electrophoresis (Hempel and Blaschke, 1996[15]), immunoassay, radioimmunoassay (Huynh et al., 2009[18]; De Clerck and Daenens, 1997[7]), GC (Winek et al., 1996[44]; Stanke et al., 1996[37]), GC-MS (Keller et al., 1999[19]; Gunnar et al., 2005[13]; Adamowicz and Kała, 2010[1]), HPLC-UV (Tracqui et al., 1993[39]; Wang et al., 1999[43]), HPLC coupled with fluorescence detection (Nirogi et al., 2006[26]; Ptáček et al., 1997[34]; Durol & Greenblatt, 1997[8]), LC-MS, and LC-MS/MS (Lewis and Vine, 2007[22]; Shi et al., 2012[35]; Giroud et al., 2003[10]; Piotrowski et al., 2015[33]; Eliassen and Kristoffersen, 2014[9]; Bhatt et al., 2006[4]) have been suggested for the determination of zolpidem in biological samples. Most of these techniques require the use of time-consuming sample treatment and expensive instrumental methods. In addition, EME has not been used for extraction of zolpidem from biological samples. To the best of our knowledge, this is the first time that EME as a new micro-extraction technique followed by a simple HPLC-UV method is developed and validated for the quantitation of zolpidem in biological samples. Major parameters associated with the efficiency of EME were evaluated and optimized. A schematic illustration of the applied process is depicted in Figure 1B(Fig. 1).

## Materials and Methods

### Chemicals and reagents

Zolpidem tartrate was supplied by Farmak (Czech Republic). Stilnox® 10 mg tablets were obtained from Sanofi Aventis (Sanofi Winthrop Industrie, France). Methanol, ammonium acetate, glacial acetic acid, hydrochloric acid, sodium hydroxide, 2-ethylhexanol, 1-heptanol, and 1-octanol were purchased from Merck (Darmstadt, Germany). The porous hollow fiber used for the supported liquid membrane (SLM) was Accurel PP 300/1200 polypropylene (inner diameter = 1200 µm, wall thickness = 300 µm, and average pore size = 0.2 µm) was prepared from Membrana (Wuppertal, Germany). Multi-walled carbon nanotubes (outer diameter <8 nm, length ∼30 μm, specific surface area >500 m^2^/g, and purity >95 %) were purchased from the Neutrino Company (Tehran, Iran). HPLC grade water was produced using a Fistreem cyclon system (Leicestershire, UK) and then was used to prepare all solutions.

### Stock, standard and biological solutions

A stock solution containing 1 mg/mL of zolpidem (base) was prepared in distilled water using ultrasonication and stored at 4 ºC while protected from the light. Sample solutions were prepared by dilution of the stock solution using distilled water. The stock solution was also used to spike human plasma and urine. Drug-free human plasma was obtained from the Iranian Blood Transfusion Organization (Tehran, Iran). Urine samples were acquired from two volunteers. The volunteers were administered a single 20 mg oral dose of zolpidem tartrate (two 10 mg stilnox® tablets) at bedtime. Then, urine samples were collected prior to and 6 hours after oral administration. All spiked biological samples were freshly prepared before the extraction.

### Modification of carbon nanotubes

In this study, carbon nanotubes (CNTs) were evaluated as nano-sorbents in SLM composition. Carbon nanotubes were used in two forms viz. pure CNTs and modified CNTs. Modification was performed by oxidation of the carbon nanotubes using previously reported procedure (Yaripour et al., 2016[45]). In order to transfer the CNTs into pores of the hollow fiber, pure CNTs and modified CNTs were dispersed in the SLM solvent at a concentration of 1 mg/mL. At the same time, they were sonicated for 20 minutes and then, 35 mm pieces of polypropylene hollow fiber were dipped into the suspension. The fiber pores were filled with the mixture by means of sonication for 10 minutes.

### HPLC conditions

HPLC analysis was performed using a previously reported method (Yaripour et al., 2015[46]) following partial modification. The modular HPLC system consisted of a Knauer HPLC Pump K-1001, a UV Detector K-2600, a Knauer injection system and a Knauer solvent degasser (Berlin, Germany). Data acquisition was performed using ChromGate® Chromatography Data System Version 3.1.7 (Berlin, Germany). AHS-5 C8 Perkin Elmer column (5 µm, 125 mm×4.6 mm i.d.) (Massachusetts, USA) was used for chromatographic separation at 24 ± 2 ºC. The mobile phase was a mixture of methanol and 50 mM ammonium acetate buffer containing 0.1 % v/v triethylamine at a pH = 3.7 which was used in a 40:60 ratio (v/v). The buffer was filtered through 0.45 µm membrane filter prior to use. A flow rate of 1 mL/min was used and UV detection was performed at 300 nm. The volume of injection was 10 µL. 

### Electromembrane extraction procedure

The DC power supply EPS-Universal model (Paya Pajohesh Pars, Tehran, Iran) with programmable voltage in the range of 0-400 V (currents of 0-0.5 A) was used to aid the EME procedure. As an EME cell, a 7 mL homemade glass tube with a screw cap was used. The glass height was 7 cm with an internal diameter of 13 mm. Platinum wires (diameter 0.2 mm) were utilized as the electrodes. An aliquot (7 mL) of the sample solution was filled in the sample extraction vial. A 35 mm piece of polypropylene hollow fiber was dipped into the organic solvent and sonicated for 1 minute to impregnate the hollow fiber. The excess organic solvent was removed from the inner and outer of hollow fiber by washing with water via a micro-syringe. The lower end of the hollow fiber was closed by a thermal and mechanical pressure and the lumen of the fiber was filled with 30 µL of the acceptor solution through a micro-syringe. The hollow fiber, including acceptor solution, was inserted into the sample solution. The positive electrode (anode) was placed in the sample solution and negative electrode (cathode) was placed directly in acceptor solution. Subsequently, the power supply was activated and then the required voltage was applied across the electrodes for a pre-defined time. During all extractions, the sample solution was stirred using a stirrer at 600 rpm. At the end of the extraction procedure, the acceptor solution was collected by a micro-syringe and analyzed by HPLC.

### Calculation of enrichment factor, extraction recovery and relative recovery 

The enrichment factor (*EF*) is defined as the ratio of the final analyte concentration in the acceptor solution (*C_a_*) to the initial concentration of analyte in the donor solution (*C_d_*) which is calculated following equation 1:


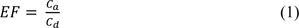


The extraction recovery (*ER*) is defined as the percentage of the number of moles of analyte originally present in the donor sample (*n_d_*), which was extracted to the acceptor solution (*n_a_*). *ER* is calculated according to equation 2:


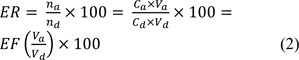


Where, *V_a_* and *V_d_* are the volumes of acceptor solution and donor sample solution.

The relative recovery (*RR*) is calculated for biological samples using equation 3:


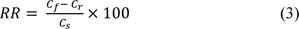


where, *C_f_* is the concentration of analyte found after addition of known amount of standard into the real sample, *C_r_* is the concentration of analyte in real sample, and *C_s_* is the concentration of known amount of standard, spiked into the real sample.

## Results and Discussion

In order to ensure the maximum extraction efficiency, the effective parameters of the EME procedure were evaluated and optimized, including membrane composition, pH of donor and acceptor solutions, applied voltage, extraction time, and carbon nanotube effect.

### Supported liquid membrane (SLM) composition

**Figure 2:**
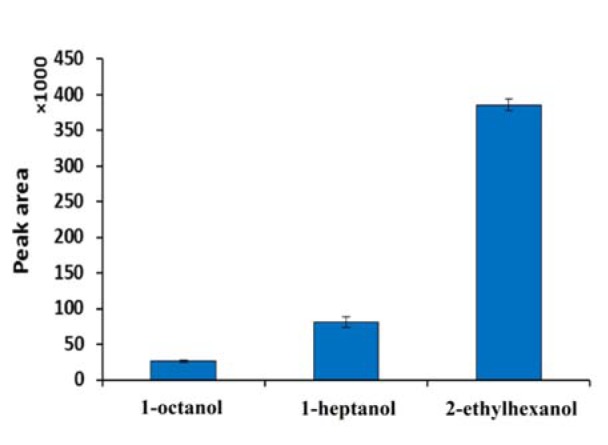
Effect of SLM composition on EME efficiency of zolpidem (extraction conditions; concentration in donor: 1 µg/mL, pH of donor: 4, pH of acceptor: 3, time of extraction: 10 min and voltage: 50 V)

The composition of membrane may affect the diffusion coefficient of the analyte of interest and therefore may influence recovery. The organic solvent used in EME should have several characteristics (Marothu et al., 2013[24]; Huang et al., 2015[17]) including adequate electrical conductivity, low vapor pressure, immiscibility in water, amongst others. Solvents such as 1-octanol, 1-heptanol, and 2-ethylhexanol were evaluated as potential components of the SLM. Other conditions of extraction were as follow: zolpidem concentration (1 µg/mL) in the donor compartment, pH of the donor fluid (pH = 4.0), pH of the acceptor fluid (pH = 3.0), the extraction time (10 min), and applied voltage (50 V). As can be seen from Figure 2(Fig. 2), 2-ethylhexanol is the most appropriate solvent which results in the most efficient extraction. In this case, 2-ethylhexanol provides stable conditions for EME in comparison to 1-heptanol and 1-octanol. It may be related to viscosity and lipophilicity of these solvents and the solvent immobilization in the pores of a hollow fiber. Lipophilicities of these three solvents are between 2.7-2.9 and they are similar to each other in lipophilicity. However, 2-ethylhexanol has the highest viscosity and exhibits a more stable SLM in this experiment in comparison to 1-octanol and 1-heptanol. By the way, the results of 2-ethylhexanol as SLM in EME procedure of basic drugs are comparable with those of nitrophenyl octyl ether (NPOE) (Asadi and Nojavan, 2016[2]) which is more toxic and expensive than 2-ethylhexanol. 

### pH of donor and acceptor solutions

**Figure 3:**
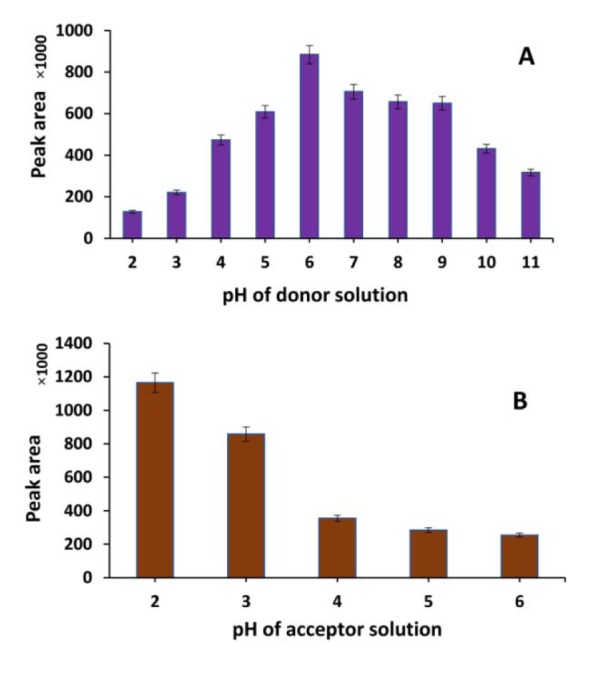
Effect of (A) pH of donor, and (B) acceptor solution on EME efficiency of zolpidem (extraction conditions; concentration in donor: 1 µg/mL, SLM: 2-ethylhexanol, time of extraction: 10 min and voltage: 50 V)

In an EME procedure, the analyte should be in the ionic form in the donor solution to ensure the maximum extraction efficiency. The pH of donor and acceptor solutions can impact the ionic balance in a system. It was revealed that the ratio of total ionic concentration in the donor solution to that in the acceptor solution has a direct impact on the flux across the membrane (Gjelstad et al., 2007[12]). Initially the effect of pH in the donor solution was evaluated whilst retaining the pH of the acceptor solution at 3.0. As shown in Figure 3A(Fig. 3), the best pH for the donor solution is 6.0. The pKa of zolpidem is 6.2 (the molecule is a weak base). Therefore in a solution with pH = 6.0, more than 50 % of molecules in the donor solution are likely ionized. Thus, a decreased pH would result in a greater ionization of molecules with a negative impact on the ionic balance in the system. It seems that increasing the HCl concentration in the donor solution leads to increasing the total ionic concentration and, consequently, enhancing ion balance in the system. Therefore, the extraction is decreased in lower pHs. The effect of pH between 2.0 and 6.0 of the acceptor solution was also evaluated whilst keeping the donor solution pH at 6.0. As found from Figure 3B(Fig. 3), the highest extraction efficiency was observed at pH = 2.0. It was observed a high variation in results of pH = 1.0 for acceptor solution. The EME system has the lowest ionic balance as well as the lowest back diffusion from the acceptor solution at pH = 2.0. In addition, pH of the donor solution was tested again, at the end of each extraction. There was no significant variation in pH of donor solution after EME. Consequently, pH of 6.0 and 2.0 were selected for the donor and acceptor solutions, respectively.

### Applied voltage

**Figure 4:**
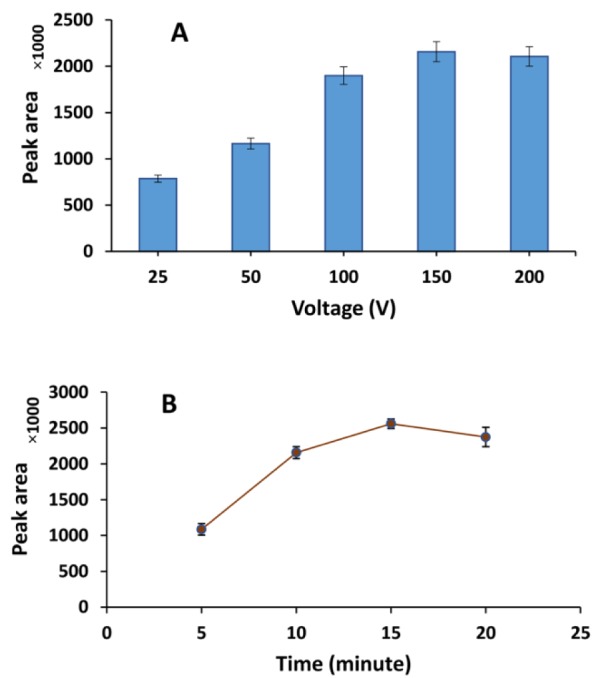
Effect of (A) voltage, and (B) extraction time on EME efficiency of zolpidem (extraction conditions; concentration in donor: 1 µg/mL, SLM: 2-ethylhexanol, pH of donor: 6, pH of acceptor: 2)

The electric potential difference between compartments is the main driving force for the migration of the analytes across liquid membranes. The applied voltage affects the flux of analytes and is one of the most important parameters that must be optimized for EME (Pedersen-Bjergaard and Rasmussen, 2006[32]; Marothu et al., 2013[24]). The extraction of zolpidem was evaluated in using applied voltage in the range 25 to 200 V. The results are depicted in Figure 4A(Fig. 4). The extractability of zolpidem was increased as the voltage was increased up to 150 V but no further enhancement was observed in the extraction with voltages >150 V. In this situation, the electric current in the system increased and resulted in electrolysis and bubble formation. These made the system unstable with a decreased efficiency of analyte/proton exchange and therefore decreased extraction efficiency. As a result, 150 V was selected as the optimum voltage and driving force for next experiments.

### Extraction time

To ensure the maximum extraction efficiency, the effect of extraction time was also assessed. As evident from Figure 4B(Fig. 4), an increase in the extraction efficiency was observed up to the extraction duration of 15 minutes after which a decrease in extraction occurred. The saturation of the analyte in the acceptor solution and the bubble formation in the donor solution are the main reasons for this phenomenon. Loss of organic solvent in the SLM as a consequence of heat generation in the system is also a possible cause of a decreased recovery (Pedersen-Bjergaard and Rasmussen, 2006[32]; Gjelstad and Pedersen-Bjergaard, 2011[11]). Therefore, 15 min was selected for the extraction time.

### Effect of carbon nanotubes

In recent years, carbon nanotubes (CNTs) have been used as nano-sorbents in extraction and purification due to their structure (electrical conductivity, high thermal stability and large available surface area). However, a few reports have employed the CNTs for EME procedures (Liang et al., 2014[23]; Hasheminasab et al., 2014[14]; Yaripour et al., 2016[45]).

In order to evaluate the effect of CNTs on an EME procedure, unmodified (pure) and modified CNTs were used in SLM composition. The results indicated that the presence of CNTs led to a decrease in the extraction efficiency, more likely due to CNTs acting as physical barriers which impede the analyte migration. CNTs exhibit electric conductivity and result in a reduced resistance of the SLM, and consequently a reduced electric field. Furthermore, the analyte may be adsorbed on the sorbents. Hence, the desorption force or applied voltage may not be sufficient to release the analyte into the acceptor solution. These phenomena probably bring about a decrease in the extraction efficiency (Yaripour et al., 2016[45]). Therefore, CNTs were not used in the SLM composition in next experiments.

### Analytical method validation

To study the performance of the proposed EME procedure for the quantitation of zolpidem, the enrichment factor, extraction recovery, linearity, repeatability, limit of detection (LOD), limit of quantitation (LOQ), and accuracy were determined using the optimized extraction conditions. The results are summarized in Table 1(Tab. 1). The enrichment factor (EF) and extraction recovery (ER) were 75 and 32, respectively. The LOD and LOQ were estimated according to a signal to noise ratio of 3 and 10, respectively. Five standard solutions were prepared for the calibration. Zolpidem was dissolved in water and the solutions were extracted using optimized conditions, and then analyzed by HPLC. The linearity was tested over the range of 10-1000 ng/mL and the resultant R^2^ value was >0.9991. The repeatability and relative error were determined at five different concentration levels and these data are summarized in Table 2(Tab. 2). The relative standard deviations (RSD%) ranged between 0.3 % and 7.3 %. The precision and accuracy data are acceptable for this EME-HPLC-UV method considering previously reported EME experimental data.

### Biological samples analysis

**Figure 5:**
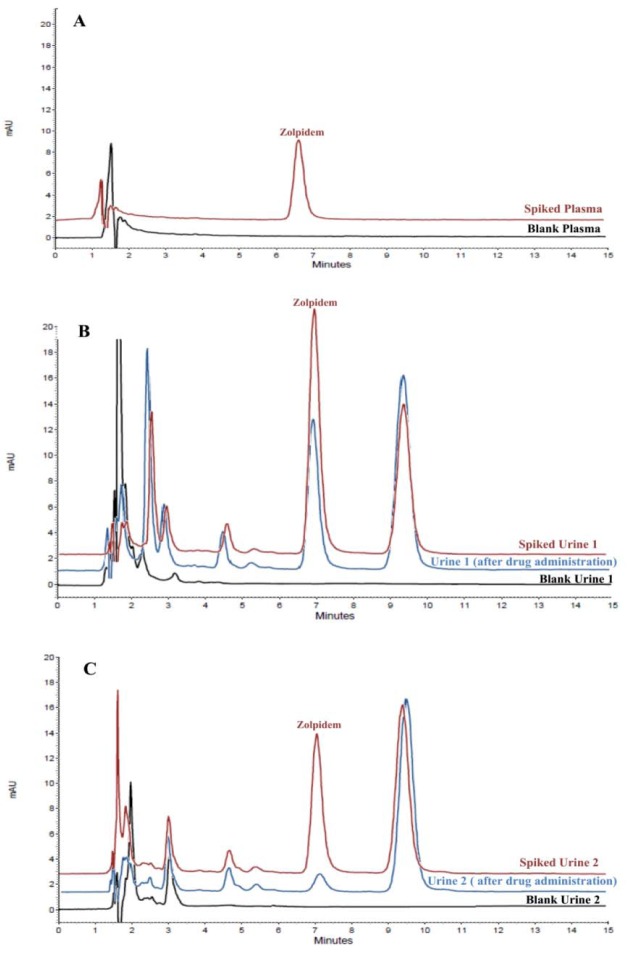
Typical chromatograms of (A) Plasma, (B) Urine of volunteer 1, and (C) Urine of volunteer 2, after EME procedure

The proposed EME method was employed for the quantitation of zolpidem in human urine and plasma samples to establish the applicability of the extraction method. Urine samples were collected from two volunteers whom had been administered zolpidem orally. In order to minimize the matrix effect, urine and plasma samples were diluted by distilled water 1:1 and 1:9, respectively, and then adjusted to pH = 6.0 before the EME process. The final concentration of zolpidem in all spiked biological samples was 100 ng/mL. As summarized in Table 3(Tab. 3), the relative recoveries were obtained between 60.3 and 78.9 % for biological samples. Presence of salts, proteins, and other macromolecules in plasma and urine led to lower relative recoveries in biological samples in comparison to the pure water. In this regard, the high protein binding of zolpidem (>92 %) can be another reason for lower recoveries in comparison to pure water. The % RSD was established following replicate (*n* = 3) analysis where the range of 2.3 to 5.5 % indicated the precision of the method for biological samples analysis. Typical chromatograms following the EME of plasma and urine samples are shown in Figure 5(Fig. 5). 

Likewise, a comparison of the proposed method to published methods for the quantitation of zolpidem from different sample matrices is listed in Table 4(Tab. 4) (References in Table 4: Wang et al., 1999[43]; Durol and Greenblatt, 1997[8]; Shi et al., 2012[35]; Giroud et al., 2003[10]; Piotrowski et al., 2015[33]; Eliassen & Kristoffersen, 2014[9]). The results suggest that the proposed method for the quantitation of zolpidem in biological samples is relevant and applicable. This technique is simple and cheap compared to methods such as LC-MS/MS and those in which complicated and time-consuming sample treatment is undertaken. In addition, the excellent separation of the target molecule from the other components in the sample matrices, including metabolites of zolpidem, is clearly visible in the urine chromatograms. It seems that the peak after zolpidem in urine chromatogram is the major urinary metabolite of zolpidem and named ZCA (zolpidem carboxylic acid) accounted for 51 % of an administered dose (Lewis and Vine, 2007[22]; Shi et al., 2012[35]; Piotrowski et al., 2015[33]). Finally, the method was directly applied to biological samples without any additional sample preparation steps. The short extraction and analysis time, consumption of small volumes of organic solvent (<15 µL) as well as a cheap and simple analytical technique, all make this method highly applicable for the analysis of zolpidem in biological matrices.

See also the Supplementary Data.

## Conclusion

An efficient and simple EME-HPLC-UV method has been reported for the determination of zolpidem in biological matrices. To extract the target molecule, parameters including SLM composition, applied voltage, pH of donor and acceptor solution, and time of extraction were evaluated and optimized. In addition, the effect of solid sorbents such as CNTs in the membrane composition and EME performance was evaluated. The EME enhances the limit of detection of the method for zolpidem and then, improves the performance of HPLC-UV for the lower concentrations of zolpidem. A short time for extraction (15 min) was provides acceptable relative recovery from biological samples. This indicates that the proposed EME-HPLC-UV method is a suitable analytical method for the quantitation of zolpidem in such matrices. 

## Acknowledgement

The authors would like to acknowledge financial and instrumental supports from Tehran University of Medical Sciences, Tehran, Iran.

## Conflict of interest

The authors declare that they have no conflict of interest.

## Supplementary Material

Supplementary data

## Figures and Tables

**Table 1:**
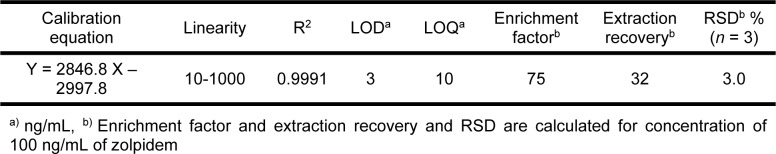
Analytical characteristics of EME-HPLC-UV analysis for zolpidem

**Table 2:**
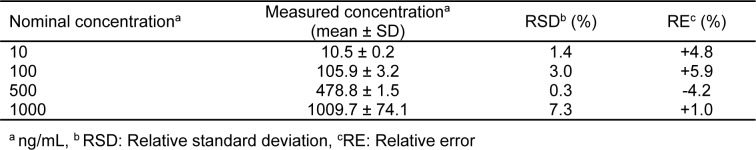
Accuracy and precision data of the proposed EME-HPLC-UV method (*n* = 3)

**Table 3:**
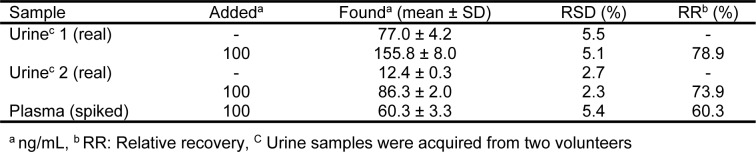
Results obtained for biological samples analysis by the proposed EME-HPLC-UV method (*n* = 3)

**Table 4:**
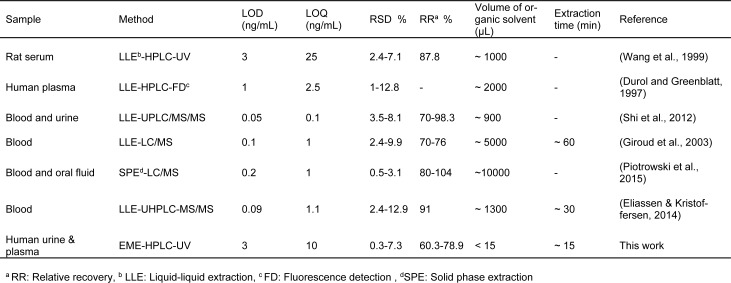
Comparison of the proposed method to published liquid chromatography methods for the quantitation of zolpidem in different sample matrices
